# Performance comparison of Gilbert’s algorithm and machine learning in classifying Bell-diagonal two-qutrit entanglement

**DOI:** 10.1038/s41598-023-46337-z

**Published:** 2023-11-09

**Authors:** Marcin Wieśniak

**Affiliations:** 1 Faculty of Mathematics, Physics, and Informatics, Institute of Theoretical Physics and Astrophysics, University of Gdańsk, 80-308 Gdańsk, Poland; 2 International Centre for Theory of Quantum Technologies, University of Gdańsk, ul. Bażyńskiego 1A, 80-309 Gdańsk, Poland

**Keywords:** Quantum physics, Quantum information

## Abstract

While classifying states as entangled or separable is one of the fundamental tasks in quantum information theory, it is also extremely challenging. This task is highly nontrivial even for relatively simple cases, such as two-qutrit Bell-diagonal states, i.e., mixtures of nine mutually orthogonal maximally entangled states. In this article we apply Gilbert’s algorithm to revise previously obtained results for this class. In particular we use “entanglement cartography” to argue that most states left in [Hiesmayr, B. C. Scientific Reports **11**, 19739 (2021)] as unknown to be entangled or separable are most likely indeed separable, or very weakly entangled, beyond any practical relevance. The presented technique can find endless applications in more general cases.

## Introduction

Entanglement is probably the most striking feature of quantum mechanics. Initially, it was introduced for pure states, as a notion that parts of a composite quantum system can be described only in reference to each other^1^. This understanding was extremely convenient, as it was immediate to recognize an entangled state, and as it turned out^2^, in the bipartite case, entanglement can be quantified uniquely as the entropy of reduced states of subsystems.

However, as the interest in foundations of quantum information theory grew, the issue got more complicated. For mixed quantum states, mathematically represented by positive semi-definite square matrices of trace 1, it is not sufficient to study the deficit of information about individual subsystems at the expense of correlations. Instead, one needs to consider if the correlations can be reconstructed by means of local operations and classical communication (LOCC). A strong indicator of presence of entanglement in a state is a negative partial transpose (NPT)^3,4^. Still, it turned out that, except for dimensions of subsystems $2\times 2$ and $2\times 3$, entangled states with positive partial transpose (PPT) exist^5^. This form of entanglement has been recognized as bound entanglement, which cannot be distilled to the form of maximally entangled states^6^, as opposed to free NPT entanglement. Technically, it remains an open question if bound entangled states with a negative partial transpose exists, but hereafter we will assume that all NPT entanglement is distillable.

More generally, all positive, but not completely positive maps were found to identify some form of entanglement^4^. Alternatively, any entangled state yields a negative mean value of a suitable entanglement witness operator, for which all separable (non-entangled) states give non-negative means (hereafter, we will adapt the opposite convention). The two methods happen to be related to each other by the Jamiołkowski-Choi isomorphism^7,8^. However, until recently there were no practical means to construct a witness tailored for a given state.

Further complications arose with attempts to give reasonable measures of entanglement. A list of requirements that any such measure must satisfy was formulated^9^ and two operational measures were defined: distillable entanglement^9^ and entanglement cost^10^. The former quantifies the number of Bell states required to prepare a copy of a given state in the LOCC, while the latter gives the number of them in the opposite process. Other measures were proposed in hope for better computability, for example based on the notion of a closest separable state (CSS)^11^, or as extensions of the measure for pure states with a convex roof extension^12^. Still, the impossibility of considering all possible state manipulation schemes, decompositions into pure states and apparent computational difficulty of computing CSS with respect to some metrics prevented all of these propositions to become universal and practical.

More modern computing techniques have also been employed to the problem of separability in specific cases. For example, there was a number of attempts to adopt machine learning techniques to distinguish entangled states from separable ones^13–25^. This approach poses its own set of challenges. First, as every use of machine learning (ML) techniques is application-specific, relatively little can be said about, e.g., the optimality of the topology of a neural network, the form of the activation function, etc.. The other issue is that the result of ML is dependent on the training data, which can be very limited for the entanglement-vs-separability problem. An algorithm can become overtrained, with a fixation on the presented pattern. Finally, neural networks are not designed to estimate the missing continuous value of a function, which makes them less capable of providing the interpolation of the amount of entanglement, although there were some attempts of such tasks^20,21^.

Recently, other strategies for classifying states as entangled or separable, based on variational algorithms^26^ and semi-definite programming^27^, were proposed.

In this article we compare the algorithmic approach described in detail below with ML results obtained by Hiesmayr^17^, who focused on “the magic simplex”, or the set of Bell-diagonal states of three qubits. The task is to classify states as free entangled, bound entangled, or separable. Our methodology is described below, followed by a presentation of results and conclusions.

## Hilbert-Schmidt distance and Gilbert’s algorithm

The Hilbert-Schmidt distance between arbitrary matrices *A* and *B* of the same size is defined simply as

1 $$\begin{aligned} D_{HS}(A,B)={} & {} \sqrt{\text {Tr}\left( (A-B)(A-B)^\dagger \right) }\nonumber \\ ={} & {} \sqrt{\sum _{j,k}\left| (A-B)_{j,k}\right| ^2}, \end{aligned}$$

with $\text {Tr}(\cdot )$ denoting the trace and $(X)_{j,k}$ denotes the element at the position (*j*, *k*) of the matrix *X*.

This figure of merit has many advantages. Obviously, it is a well-defined distance, being just a variant of the usual Euclidean metric. It also allows us to use intuitive terms from everyday geometry, such as spheres, angles, or intervals. It is also the only measure, which can be calculated without a computationally expensive subroutine for singular decomposition of a matrix.

Unsurprisingly, it was suggested as a core of an entanglement measure by Witte and Trucks^28^. For state $\rho$ this entanglement quantifier shall be defined as

2 $$\begin{aligned} D_{HS}(\rho )=\min _{\sigma \in SEP}D_{HS}(\rho ,\sigma ), \end{aligned}$$

where the minimum is taken over all separable states. However, it was shown by Ozawa^29^ that the Hilbert-Schmidt distance does not comply with the requirement of contractivity under a local operation applied to both states, although it remains unclear if it holds for the minimaliozation. Other complications may occur when migrating a state to another Hilbert space by adding or tracing out an auxiliary system. Still, given the advantages of the Hilbert-Schmidt measure, it can be seen as an insightful quantifier of non-classical correlations.

In 1966, Gilbert^30^ introduced an algorithm to estimate the distance between a given point and a convex set. It was demonstrated to be useful to show the membership of a state in an LOCC equivalence class^31^, and to optimize Bell operators^32^. Finally, Pandya, Sakarya, and Wieśniak^33^ discussed a direct use of the algorithm to estimate the distance of a given quantum state to the set of separable states. The algorithm can be outlined as follows.

### Algorithm 1

(bipartite case):

*Input:* test state $\rho _0$, initial separable state $\rho _1$.

* Output:* approximation of CSS $\rho _1$, list of squared distances to subsequent CSS approximations *l*.

1. Take random pure state $\rho _2=\left| \varphi _A\right\rangle \left| \varphi _B\right\rangle \left\langle \varphi _A\right| \left\langle \varphi _B\right|$, that will be referred to as a trial state (run Algorithm 2).
2. If the preselection criterion, $\text {Tr}(\rho _0-\rho _1)(\rho _2-\rho _1)>0$ is not met, go to step 1 or abort if the HALT condition is satisfied.
3. Maximize $\text {Tr}(\rho _0-\rho _1)(\rho _2-\rho _1)$ with local unitary transformations (run Algorithm 3).
4. Update $\rho _1\leftarrow p \rho _1+(1-p)\rho _2$ for *p* minimizing $D_{HS}(\rho _0,p \rho _1+(1-p)\rho _2)$.
5. Every 50 corrections append $D^2_{HS}(\rho _0,\rho _1)$ to *l*.
6. If the HALT condition is not met, go to step 1, otherwise quit.

Pure random states are generated in the following subroutine^34^.

### Algorithm 2

:

*Input:* dimension *d*.

* Output:* pure *d*-dimensional state randomly generated with respect to the Haar measure $\left| \psi \right\rangle$.

1. Draw 2*d* random numbers $r_1,r_2,...,r_{2d}$ from a normal distribution centered at 0.
2. Construct $$\left| \psi \right\rangle =\{c_j\}_{j=1}^d=\{r_{2j-1}+i r_{2j}\}_{j=1}^{d}$$.
3. Normalize $\left| \psi \right\rangle$ so that $\left\langle {\psi }|{\psi }\right\rangle =1$.

Hereafter, *i* will denote the imaginary unit, $i^2=-1$.

Features and performance of Algorithm 1 have been studied in Ref.^33^, with two major updates introduced in this work. One of them is the optimization in Step 3, which, depending on $\rho _0$, can speed up the convergence up to approximately 50 times, in terms of the required number of trials. In the code used for this article the optimization was performed with the following algorithm.

### Algorithm 3

:

*Input:* states $\rho _0$, $\rho _1$ and $\rho _2$.

*Output:* optimized state $\rho _2$.

For $$j=1$$ to 1500:

do

1. draw a pure random qutrit state $\left| \psi \right\rangle$ uniformly with respect to the Haar measure (run Algorithm 2).
2. With *j* odd construct
  3 $$\begin{aligned} U=\left( \mathbbm {1}+\left( e^{i\pi /100}-1 \right) \left| \psi \right\rangle \left\langle \psi \right| )\right) \otimes \mathbbm {1}. \end{aligned}$$
  With *j* even construct
  4 $$\begin{aligned} U=\mathbbm {1}\otimes \left( \mathbbm {1}+ \left( e^{i\pi /100}-1 \right) \left| \psi \right\rangle \left\langle \psi \right| )\right) . \end{aligned}$$
3. If $$\text {Tr}(\rho _0-\rho _1)(\rho _2-\rho _1)>\text {Tr}(\rho _0-\rho _1) \left( U\rho _2U^\dagger -\rho _1 \right) $$, replace $$U\leftarrow U^\dagger $$
4. replace $$\rho _2\leftarrow U\rho _2U^\dagger $$ until $$\text {Tr}(\rho _0-\rho _1)(\rho _2-\rho _1)>\text {Tr}\left( \rho _0-\rho _1 \right) \left( U\rho _2U^\dagger -\rho _1 \right) $$

done.

The algorithm provides three pieces of information that can be used here to classify states as entangled or separable. The first is, rather trivially, the last squared distance $D^2_{\text {Last}}$ found within a fixed number of corrections. For systems of this size, the distance obtained after more than 1000 corrections is believed to be a fairly good representation of the actual distance. In fact, some separable states can be reconstructed nearly down to the numerical precision in a few corrections, and are therefore safely assumed to be separable. As the algorithm in principle cannot reach $D_{\text {Last}}^2=0$, this indicator tends to overestimate entanglement for states close the entangled-separable boundary. Our approach puts the emphasis on the quantitative, rather than qualitative description of entanglement.

The second indicator is the distance decay estimate, $D^2_{\text {Est}}$. This is the other improvement with respect to Ref.^33^. The current recommendation is to maximize the linear regression coefficient,

5 $$\begin{aligned} R(x,y)=\frac{\text {Cov}(x,y)}{\sqrt{\text {Var}(x)\text {Var}(y)}}, \end{aligned}$$

with Cov and Var denoting covariance between the list of correction numbers *c* and $1/(l-a)$ (each element of *l* is shifted by *a* and then inverted), and the variances of these lists. We reject the first one third of the entries, after which the lists are denoted by $\tilde{\cdot }$. We thus have

6 $$\begin{aligned} D_{\text {Est}}^2=\text {argmax}_a R \left( \tilde{c},1/\left( \tilde{l}-a \right) \right) ,\,0\le a\le \min l. \end{aligned}$$

Here, $\text {argmax}_xf(x)$ yields the value of *x* that maximizes *f*(*x*). Although, unlike the other two indicators, $D^2_{\text {Est}}$ does not certify anything, but is merely an result of a statistical analysis, we shall find it to be quite informative. With $R(x,e x+f)=1$, $e>0$ and *f* being real numbers, we typically get $R\approx 0.998$ for 10,000 corrections.

One advantage of $D_{\text {Est}}$ over $D_{\text {Last}}$ is that the distance decay estimate can be found to be 0, suggesting no entanglement in the studied state.

The third and final figure of merit considered here is the witness distance estimate $D_{\text {Wit}}$. As Ref.^35^ points out, if $\rho _{0,\text {CSS}}$ is the actual closest separable state, operator $W_0=\rho _0-\rho _{0,\text {CSS}}$ attains positive expectation values only for entangled states, thus being an entanglement witness. In reality, $\rho _1$ is only an approximation of $\rho _{0,\text {CSS}}$, displacing and tilting the hyperplane represented by the witness. As stated in Ref.^36^, we then need to consider

7 $$\begin{aligned} W=\rho _0-\rho _1-\mathbbm {1}\max _{\left| \phi _1\right\rangle ,\left| \phi _2\right\rangle }\left\langle \phi _1\right| \left\langle \phi _2\right| (\rho _0-\rho _1)\left| \phi _1\right\rangle \left| \phi _2\right\rangle , \end{aligned}$$

with the maximum taken over all product states. Then

8 $$\begin{aligned} D_{\text {Wit}}=\max \left( 0,\frac{\text {Tr}W\rho _0}{\sqrt{\text {Tr}(\rho _0-\rho _1)^2}}\right) . \end{aligned}$$

Again, this quantity depends on the quality of the approximation $\rho _1$, and provides a bound, this time lower, on the actual distance. It is the only one, out of the three quantities, that actually certifies the presence of entanglement. However, calculating it is more challenging computationally than it seems. To claim to have found the global maximum of a trigonometric polynomial we need to have a high confidence level that we did not obtain a value for any local extrema. This is done, for example, by multiple repetitions of the maximization with randomized initial conditions. Having taken care of it, we shall assume that we have indeed found the absolute maximum.

## “Magic Simplex” states

Equipped with the above tools to analyse entanglement, we will revisit certain classes of two-qutrit Bell diagonal states, also known as “Magic Simplex” states.

Let us consider a maximally entangled state of two qutrits,

9 $$\begin{aligned} \left| \psi _{00}\right\rangle =\frac{1}{\sqrt{3}}\sum _{j=0}^2\left| jj\right\rangle , \end{aligned}$$

and two Weyl operators, $$X=\left( \begin{array}{ccc}0&{}1&{}0\\ 0&{}0&{}1\\ 1&{}0&{}0\end{array}\right) $$ and $$Z=\left( \begin{array}{ccc}1&{}0&{}0\\ 0&{}\alpha &{}0\\ 0&{}0&{}\alpha ^2\end{array}\right) $$ with $\alpha =e^{2i\pi /3}$. The Bell basis is given by

10 $$\begin{aligned} \left\{ \left| \psi _{jk}\right\rangle \right\} _{j,k=0}^2=\left\{ \mathbbm {1}\otimes X^jZ^k\left| \psi _{00}\right\rangle \right\} _{j,k=0}^2. \end{aligned}$$

Then the Bell-diagonal states are

11 $$\begin{aligned}{} & {} \rho =\sum _{j,k=0}^2p_{jk}\left| \psi _{jk}\right\rangle \left\langle \psi _{jk}\right| ,\nonumber \\{} & {} \sum _{j,k=0}^2p_{jk}=1, p_{jk}\ge 0. \end{aligned}$$

In particular, Hiesmayr focused on four families of states. Family *A* was given by

12 $$\begin{aligned} \rho (\alpha ,\beta ,\gamma )={} & {} (1-\alpha -\beta -\gamma )\frac{\mathbbm {1}}{9}\nonumber \\{} & {} \quad +\alpha \left| \psi _{00}\right\rangle \left\langle \psi _{00}\right| \nonumber \\{} & {} \quad +\beta \left| \psi _{01}\right\rangle \left\langle \psi _{01}\right| \nonumber \\{} & {} \quad +\gamma \left| \psi _{02}\right\rangle \left\langle \psi _{02}\right| , \end{aligned}$$

and it was considered for $\gamma =0$. The other three families are a part of Family *B*, which includes all the states in form

13 $$\begin{aligned} \rho (\alpha ,\beta ,\gamma ,\delta ){} & {} =\left( 1-\alpha -\beta -\gamma -\delta \right) \frac{\mathbbm {1}}{9}\nonumber \\{} & {} \quad +\alpha \left| \psi _{00}\right\rangle \left\langle \psi _{00}\right| \nonumber \\{} & {} \quad +\frac{\beta }{2}(\left| \psi _{01}\right\rangle \left\langle \psi _{01}\right| +\left| \psi _{02}\right\rangle \left\langle \psi _{02}\right| )\nonumber \\{} & {} \quad +\frac{\gamma }{3}\sum _{j=0}^2\left| \psi _{1j}\right\rangle \left\langle \psi _{1j}\right| \nonumber \\{} & {} \quad +\frac{\delta }{3}\sum _{j=0}^2\left| \psi _{2j}\right\rangle \left\langle \psi _{2j}\right| . \end{aligned}$$

This family was studied in three different cases. Family $B_1$ had $\gamma =-\,\frac{1}{\sqrt{3}}$ and $\delta =0$, Family $B_2$ is parameterized by $\gamma =-0.83$ and $\delta =0$, while $B_3$ consisted of states with $\alpha =\frac{5}{3}\left( \sqrt{3}-1 \right)$ and $\beta =-\,\frac{1}{10}$.

The aim of this work is to recover, or possibly expand on results presented recently by Hiesmayr in Ref.^17^ and to verify the prediction of the machine learning presented therein. For selected families, first, the states were checked for negative partial transposition identifying all the states containing free entanglement (FREE). For the rest, which could be either bound entangled (BOUND) or separable (SEP), other known criteria of entanglement were collected. Another portion of states was recognized as bound entangled in a numerical search of respective entanglement witnesses, reportedly being the most computationally demanding part. Finally, neural networks were trained to classify remaining unknown (UNKNOWN) states as SEP or BOUND. This, however, required pre-assigning a class to each state, to be changed later by the classification algorithms.

These results were later improved to a classification of all Bell-diagonal two-qutrit states^37^ with probability of about 95$\%$. However, Ref.^37^ only provides volumetric estimates of entanglement, while Gilbert’s algorithm gives more information on the distance.

## Results

In this work we utilize a technique of entanglement cartography. For randomly scattered states within a given set, we calculate $D_{\text {Last}}$, $D_{\text {Est}}$, and $D_{\text {Wit}}$. The found values are then interpolated, and the resulting charts can be compared with plots in Ref.^17^. The charts contain evidence, if not solid proofs, for the boundaries of BOUND and SEP regions.

### Family A

**Figure 1:**
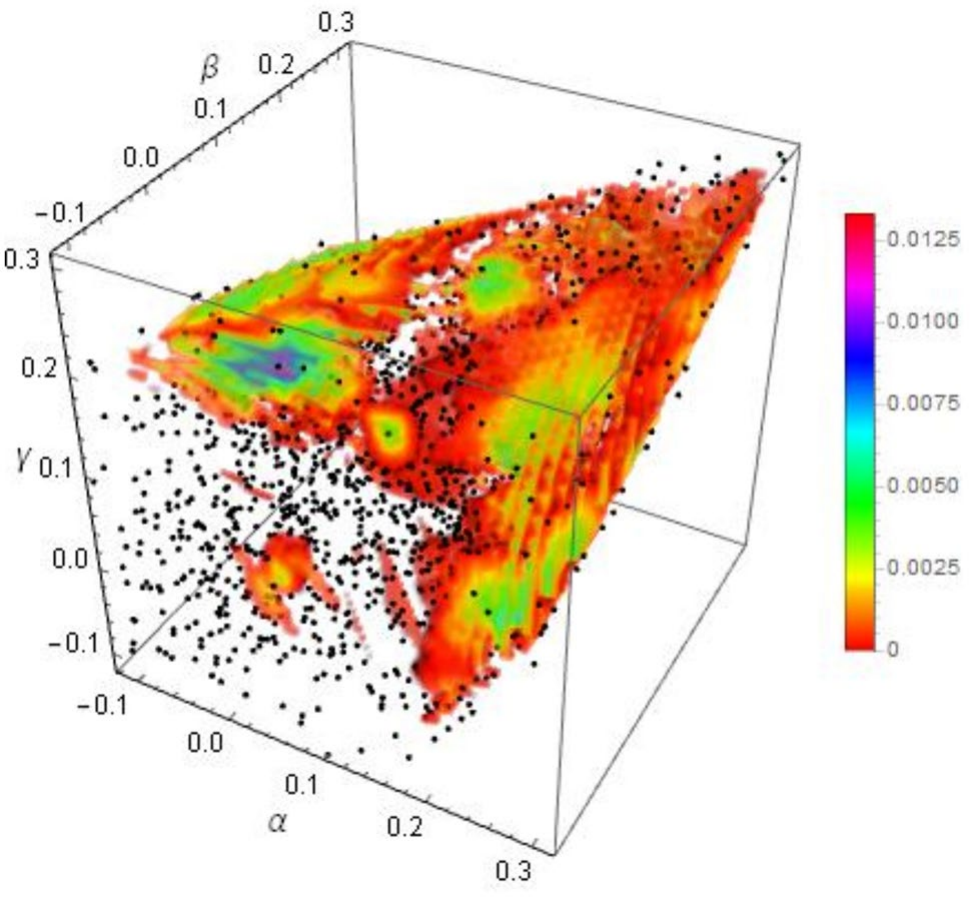
3D plot of $D_{\text {Last}}$ for PPT states from family A. Black dots represent studied states.

We first focus on Family *A*, in which we studied 1485 PPT states, each with up to 4000 corrections (the algorithm HALTS at $D_{HS}^2(\rho _0,\rho _1)<10^{-7}$). Figure 1 presents a 3D plot of $D_{\text {Est}}$. It presents an almost hollow structure. This is due to the plot renderer omitting the extreme values for clarity and the convergence being slower at the boundary between separable and entangled states. The only prominent maxima correspond to small sets bound entangled states confirmed in Refs.^38,39^. No entanglement witnesses were found. It is thus reasonable to assume that for this family bound entanglement exists only in neighbourhoods found in Refs.^38,39^.

#### Close-up

**Figure 2:**
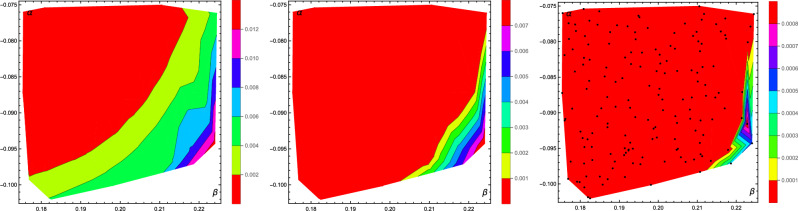
The interpolated plots of $D_{\text {Last}}$ (left), $D_{\text {Est}}$ (center), and $D_{\text {Wit}}$ (right) for Family *A* with $\gamma =0$ (only a region with PPT entangled state). Black dots represent studied states.

We have thus performed a more detailed study of 160 Family *A* PPT states with $\alpha \in (0.175,0.225)$, $\beta \in (-\,0.075,-\,0.025)$, and $\gamma =0$. We started with 90,000 corrections for each state, and extended the study for six states, which had $D_{\text {Est}}>0$. However, within HALT condition of up to 220000 corrections or 2 billion trial states we have found only three states with $D^2_{\text {Wit}}>0$. We thus leave the issue of the remaining three states unresolved by our method. Nevertheless, we remark that the plot for $D_{\text {Est}}$ reproduces the shape of the set of bound entangled states presented in Figuer 3 of Ref.^17^. It should be also noted that for the eight states with $D_{\text {Est}}>0$ the regression coefficient was greater than 0.9999. The results for $D_{\text {Last}}$, $D_{\text {Est}}$, $D_{\text {Wit}}$ are presented in Fig. 2.

### Family *B*

**Figure 3:**
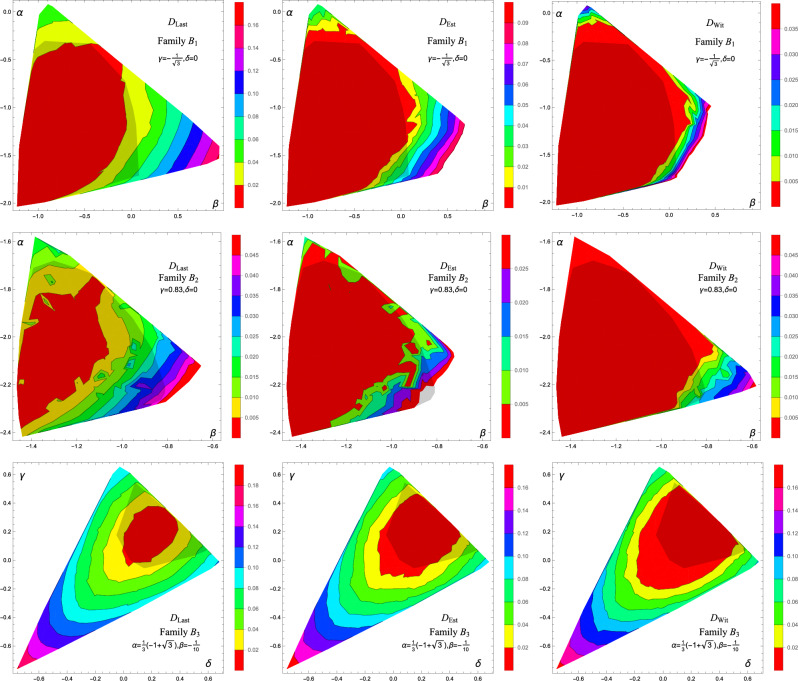
The interpolated plots of $D_{\text {Last}}$ (left), $D_{\text {Est}}$ (center), and $D_{\text {Wit}}$ (right) for Families $B_1$ (top), $B_2$ (middle) and $B_3$ (bottom). Shaded areas represent PPT states.

Subsequently, we have conducted the analysis for the remaining three families. Within each family we generated 300 states. For Family $B_1$, $B_2$, and $B_3$ we conducted up to 10,000, 8000 and 10,000 corrections respectively. The results are presented in Fig. 3 and they seem to adequately reproduce the plots in Ref.^17^. Optimization for states belonging to Family $B_2$ was significantly slower, meaning that they require significantly more trial states for a correction. Hence, the algorithm was HALTed with fewer corrections.

#### Close-up of Family $B_3$

**Figure 4:**
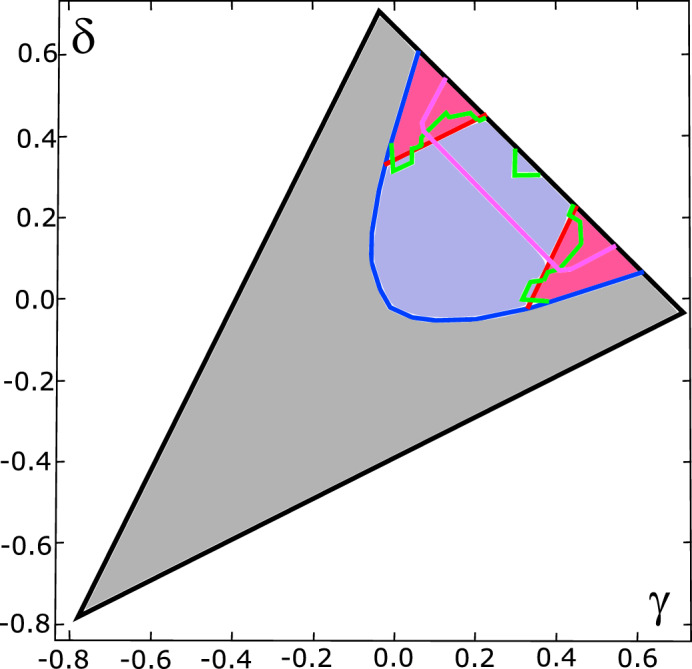
Classification of Family $B_3$ according to Ref.^17^. The color coding is as follows: gray: free entangled states, red: bound entangled states confirmed by linear witnesses, green and magenta lines: boundaries of the set of PPT entangled states as suggested by machine learning, with “nearest neighbours” and “random foresting”, respectively.

**Figure 5:**
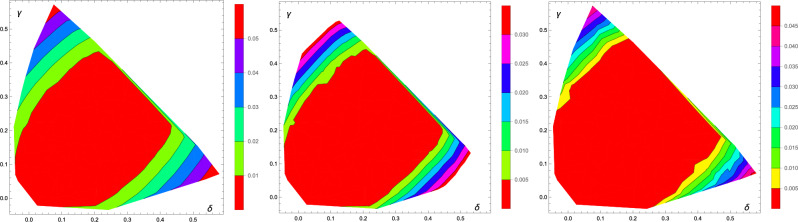
Interpolated plots of $D_{\text {Last}}$ (left), $D_{\text {Est}}$ (center), and $D_{\text {Wit}}$ (right) for PPT states of Family $B_3$.

However, we have performed an extensive study of the PPT part of Family $B_3$. This Family was chosen primarily because only for families $B_2$ and $B_3$ Hiesmayr presented machine learning results with two different preassignment strategies, “random forest” and “nearest neighbours”. For Family $B_2$, one of the results suggests all states to be entangled, and both results contradict our findings. For Family $B_3$ the two approaches gave rather inconsistent results, which are aggregated in Fig. 4. We have generated 160 states, which were studied up to 60,000 corrections each. This number of corrections provided very high consistency between $D_{\text {Last}}$, $D_{\text {Est}}$, and $D_{\text {Wit}}$. We found 32 entanglement witnesses. The results are presented Fig. 5. Importantly, the set of states recognized as entangled exceed the set bounded by witnesses presented in Ref.^17^. In this case, the algorithm found a new set of bound entangled states. For example, a state with coordinates $(\gamma ,\delta )=(-\,0.0226, 0.3067)$ was confirmed to be entangled while it was not detected in the original Reference. Also, the boundary between bound entangled and separable states seems to be curved, rather than straight. Given the geometric interpretation of the problem, this is actually expected.

#### Finer study of Family $B_2$

**Figure 6:**
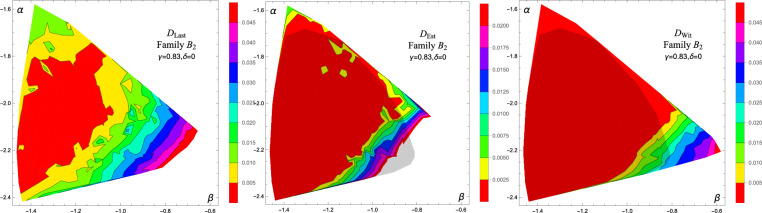
Interpolated plots of $D_{\text {Last}}$ (left), $D_{\text {Est}}$ (center), and $D_{\text {Wit}}$ (right) for PPT states of Family $B_2$ with up to 40,000 corrections for selected states.

We also selected 34 states from Family $B_2$ that are close to an expected boundary between separable and entangled states. We carried the calculation up to 40,000 corrections. The results eliminated much of the chaos visible in the middle row of Fig. 3, but still the region of $D_{\text {Wit}}>0$ is smaller than as presented in Ref.^17^ (see Fig. 6).

### Volumetry of bound entanglement in the simplex

In each case Hiesmayr gave a detailed estimate of the relative volume of bound entangled states. In this paper we rather avoid it due to substantial differences in methodology. We do not perform a massive survey of states, but take a small sample and interpolate the results. Additionally, while the algorithm is capable of precisely estimating the distance for highly entangled states and deeply separable states, it leaves many boundary cases undecided. Moreover, we use three different estimators, each dependent on how far we go in the endless sequence of corrections. These arguments thus draw the relative volumes somewhat arbitrary. The only case where we decided to investigate this issue was the full simplex parameterized by

14 $$\begin{aligned} \rho ={} & {} \sum _{(j,k)\ne (2,2)}p_{jk}\left| \psi _{jk}\right\rangle \left\langle \psi _{jk}\right| \nonumber \\ +{} & {} \left( 1-\sum _{(j,k)\ne (2,2)}p_{jk}\right) \left| \psi _{22}\right\rangle \left\langle \psi _{22}\right| . \end{aligned}$$

With probabilities $\{p_{jk}\}_{(j,k)\ne (2,2)}$ drawn at random uniformly, we generated 1000 PPT states, which took the total of 2456 physical states. This gives the ratio of 0.407 of states being PPT We run the algorithm up to 30,000 corrections for each state and found that $D_{\text {Est}}$ was larger than 0 for 137 states, whereas $D_{\text {Wit}}>0$ was found in 45 cases. We have also checked the following parameterization including white noise,

15 $$\begin{aligned} \rho ={} & {} \sum _{j,k=0}^2a_{jk}\left| \psi _{jk}\right\rangle \left\langle \psi _{jk}\right| \nonumber \\{} & {} \quad +\left( 1-\sum _{j,k=0}^2a_{jk}\right) \frac{\mathbbm {1}}{9}, \end{aligned}$$

where each $a_{jk}\in \{-1/8,1\}$ was drawn randomly with a uniform distribution. Generating 1000 PPT states took 2398 physical states, which translates into 0.417 states being PPT. Again, we have reconstructed CSSs up to 30,000 corrections. As a result, we found 139 cases of $D_{\text {Est}}>0$ and 45 cases of $D_{\text {Wit}}>0$.

### Dynamics of $D_{\text {Est}}$

**Figure 7:**
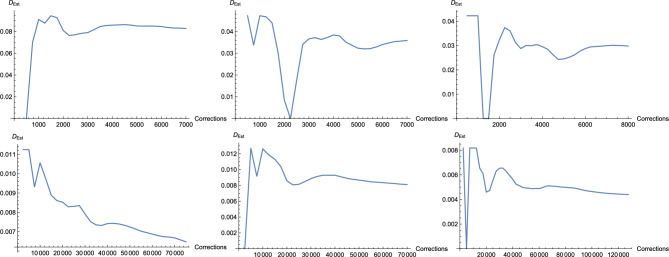
Dynamics of $D_{\text {Est}}$ in function of the number of corrections for three arbitrarily selected states from Family *A* (top) and *B* (bottom).

We also present the dynamics of $D_{\text {Est}}$ in function of the number of corrections. Figure 7 depicts this quantity for three arbitrarily chosen states from Family *B* and three from the closeup of Family *A* (Fig. 2). The only criterion was that $D_{\text {Est}}$ is larger than 0. While it is difficult to characterize this behavior in general, one can conclude that for many states $D_{\text {Est}}$ stabilizes after sufficiently many corrections. This again highlights the relevance of this quantity as an entanglement estimator.

## Conclusions

Until recently, even a rough classification of quantum states as separable or entangled had been a very demanding challenge, close to impossible. The Gilbert algorithm does not give a definite and final solution to this problem, but it can provide useful insight. First, it is able to provide incredibly precise approximations of some states, strongly hinting them as separable. On the other hand, it provides entanglement witnesses, directly certifying non-separability. This leaves few ambiguous cases of very weakly entangled states and those, for which subsequent corrections require particularly many trial states. This was the case of Family $B_2$. In this contribution we have demonstrated that a strictly statistical interpretation of the result utilizing $D_{\text {Est}}$ can also give a reasonable approximation of a set of separable states. In at least one case we have certified entanglement where other combined analytical, numerical, and machine learning-based techniques have failed.

Let us stress the low hardware requirements of the algorithm. A typical computation time for a single state with up to 10,000 corrections was around 20 min on an Intel Core i9-11900K processor (running sixteen single-core instances) and all instances together utilized far less than 1 GB of RAM at any time. It is thus both computationally and resource-wise efficient.

The presented technique of “entanglement cartography” can be universally applied to identify or estimate the boundary between separable and entangled states, regardless of the dimension, the number of subsystems, or a type of quantum correlations in question. For example, it can find a variety of applications in solid state models. Importantly, the algorithm has not been fed with any information other than the input state. It is irrelevant if the state has free or bound entanglement. The technique could be combined with machine learning and interpolation techniques, but it can also generate useful results on its own. In contrast to FREE/BOUND/SEP categorization, it provides a qualitative information about entanglement. While new bound entangled states can be easily detected, the question is if their non-classicality can be meaningful in an experimental realization.

## Data Availability

The source code is available at https://www.github.com/wiesnim9/CSSFinder. The data are available from the Author upon a reasonable request at marcin.wiesniak@ug.edu.pl.
